# Indoor formaldehyde removal over CMK-3

**DOI:** 10.1186/1556-276X-7-7

**Published:** 2012-01-05

**Authors:** Hyung Bum An, Mi Jin Yu, Ji Man Kim, Mingshi Jin, Jong-Ki Jeon, Sung Hoon Park, Seung-Soo Kim, Young-Kwon Park

**Affiliations:** 1 Graduate School of Energy and Environmental System Engineering, University of Seoul, Seoul 130-743, South Korea; 2 Department of Chemistry, BK21 School of Chemical Materials Science and Department of Energy Science, Sungkyunkwan University, Suwon 440-746, South Korea; 3 Department of Chemical Engineering, Kongju National University, Cheonan 331-717, South Korea; 4 Department of Environmental Engineering, Sunchon National University, Suncheon 540-742, South Korea; 5 Department of Chemical Engineering, Kangwon National University, Samcheok 245-711, South Korea; 6 School of Environmental Engineering, University of Seoul, Seoul 130-743, South Korea

**Keywords:** low-concentration formaldehyde, mesoporous carbon, sulfuric acid, ammonia, activation, adsorption

## Abstract

The removal of formaldehyde at low concentrations is important in indoor air pollution research. In this study, mesoporous carbon with a large specific surface area was used for the adsorption of low-concentration indoor formaldehyde. A mesoporous carbon material, CMK-3, was synthesized using the nano-replication method. SBA-15 was used as a mesoporous template. The surface of CMK-3 was activated using a 2N H_2_SO_4 _solution and NH_3 _gas to prepare CMK-3-H_2_SO_4 _and CMK-3-NH_3_, respectively. The activated samples were characterized by N_2 _adsorption-desorption, X-ray diffraction, and X-ray photoelectron spectroscopy. The formaldehyde adsorption performance of the mesoporous carbons was in the order of CMK-3-NH_3 _> CMK-3-H_2_SO_4 _> CMK-3. The difference in the adsorption performance was explained by oxygen and nitrogen functional groups formed during the activation process and by the specific surface area and pore structure of mesoporous carbon.

## Introduction

Currently, people spend more than 80% of their daily life indoors and are exposed to serious health risks due to indoor air pollution. In particular, the increased airtightness required for energy saving results in the accumulation of pollutants in less-ventilated indoor air. Sick building syndrome causing throat and nasal pains, headache, nausea, and vomiting due to indoor air pollution has become an important social issue with the increasing desire to improve the quality of life [1-3].

Formaldehyde is a representative indoor pollutant that is emitted from indoor furniture paint and floor materials. Formaldehyde has been categorized as a group 1 carcinogen by the International Agency for Research on Cancer [4]. Therefore, technology for removing formaldehyde is of great importance [5].

Adsorption, scrubbing, and advanced oxidation have been applied to remove volatile organic compounds, such as formaldehyde. In particular, adsorption using activated carbon is a method used most widely for removing formaldehyde [6]. Although the adsorption performance of activated carbon is excellent, it is not very efficient for the adsorption of polar species, such as formaldehyde. Therefore, research is being carried out to develop more efficient adsorbents for formaldehyde [7].

Recently, the synthesis and application of ordered mesoporous carbons with a variety of structures, e.g., CMK-1, CMK-3, and CMK-5, have attracted considerable attention [8-11]. These ordered mesoporous carbons have been synthesized by carbonizing mesoporous silica materials, such as MCM-48, SBA-15, and KIT-6, and then by removing the silica template. Ordered mesoporous carbon is expected to have an extensive potential in a range of applications because of the uniform pore size, large specific surface area, and large pore volume [12]. These materials are considered to have a potential for applications to other fields, such as heterogeneous catalysis and host-guest chemistry [13-16]. In particular, CMK-3, based on SBA-15, which is easy to synthesize, is expected to be useful not only as an adsorbent but also as a catalyst substrate [12].

In this study, CMK-3 was applied for the first time to the adsorption of formaldehyde. The effect of modifying the surface of CMK-3 via a range of activations to improve the formaldehyde removal efficiency was evaluated.

## Experimental details

### Synthesis of CMK-3

An ordered mesoporous carbon, CMK-3, was prepared using the nano-replication method. Mesoporous silica SBA-15 was used as a template. A carbon precursor solution was prepared by dissolving 1.25 g of sucrose in a mixture containing 4 g of deionized water and 0.14 g of H_2_SO_4_. The solution was allowed to infiltrate into the mesopores of the silica template. The mixture was dried at 100°C for 6 h. The impregnation and drying procedures were repeated twice using 66% sucrose. The carbonization was carried out at 900°C for 3 h under a nitrogen flow. Finally, CMK-3 was obtained by removing the silica matrix using an HF solution.

Ammonia and sulfuric acid treatments were applied to upgrade the characteristics of CMK-3. The ammonia treatment was performed by inserting CMK-3 into a reactor maintained at 900°C and allowing ammonia gas to flow through the reactor at a flow rate of 50 ml/min for 2 h. The reactor was purged for another 1 h with nitrogen gas at a flow rate of 50 ml/min. CMK-3, which was treated with ammonia in this way, is referred to as CMK-3-NH_3_.

The method of the sulfuric acid treatment of CMK-3 is as follows: CMK-3 was immersed in a 2N sulfuric acid solution prepared by using 95% sulfuric acid. The solution was stirred for 3 h at room temperature. The solution was then filtered, and the filtered sample was immersed in 100 g of distilled water and stirred for 1 h. This filtration and washing procedure was repeated at least ten times to neutralize the sample. The washed sample was dried for 24 h in an oven maintained at 110°C. The CMK-3 treated with sulfuric acid is referred to as CMK-3-H_2_SO_4_.

### Characterization of mesoporous carbons

X-ray diffraction [XRD] was carried out in reflection mode using a Rigaku D/MAX-2200 Ultima diffractometer (Rigaku International Corporation, Tokyo, Japan) equipped with Cu Kα radiation at 30 kV and 40 mA. N_2 _adsorption-desorption isotherms were collected on a Micromeritics Tristar system (Micromeritics Instrument Corporation, Chiba, Japan) at liquid N_2 _temperature. The specific BET surface area was calculated from the adsorption branches in the relative pressure (P/P_0_) range of 0.05 to 0.20. The pore size distribution curves were obtained from the adsorption branches using the BJH method.

X-ray photoelectron spectroscopy [XPS] measurements were performed using an AXIS Nova spectrometer (Kratos. Inc., NY, USA). A monochromatic Al Kα (1,486.6 eV) of X-ray source and 40 eV of analyzer pass energy were used under ultra-high vacuum conditions (5.2 × 10^-9 ^Torr).

### Adsorption of formaldehyde

The adsorption experiments were carried out using the method described in the literature [17]. The 100-ppm formaldehyde produced by Union Inc. was used in this study. A 10-L aluminum bag (to prevent oxidation by sunlight) was cleaned with N_2 _gas and emptied using a vacuum pump. After introducing formaldehyde into the bag, N_2 _gas was added to adjust the formaldehyde concentration to 1 ppm. Subsequently, 0.07 g of mesoporous carbon, which had been dried in advance for 24 h in an oven maintained at 100°C to remove the influence of moisture, was inserted into the aluminum bag. The temperature was controlled at 30°C using an incubator during the adsorption process to avoid any temperature dependency [17]. The sample bags were agitated in a shaking incubator to promote gas-solid mixing.

The amount of formaldehyde adsorbed at 0, 10, 40, and 80 min was measured using a formaldehyde analyzer (4000Series; Woori Industrial System Co., Ltd., Cheongwon-gun, South Korea).

## Results and discussion

### Characterization of mesoporous carbons

**Figure 1:**
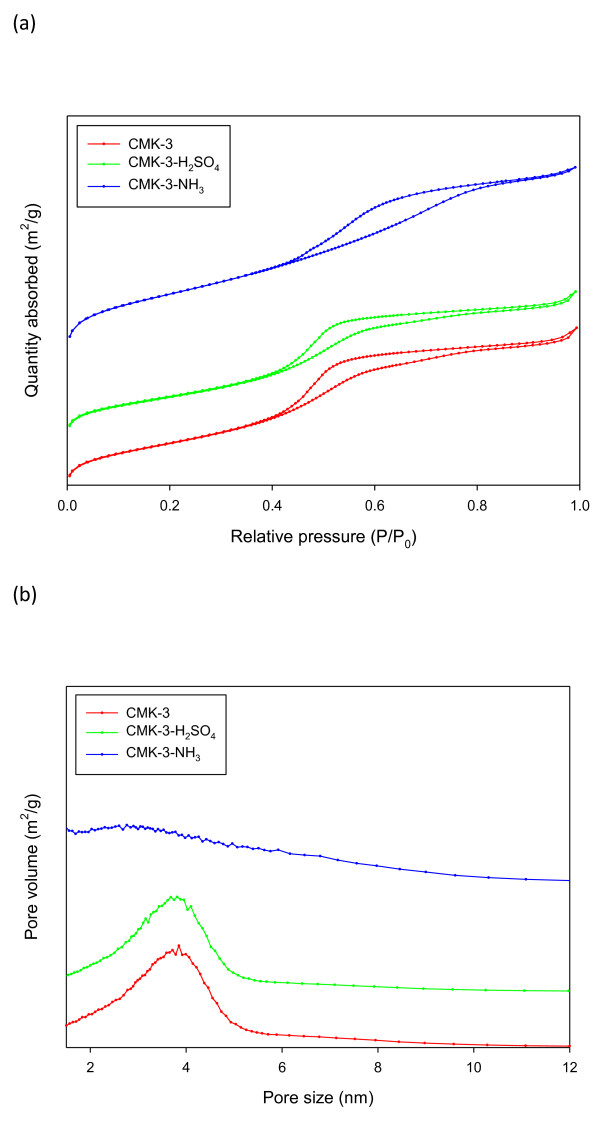
**Adsorption-desorption isotherms and pore size distribution**. (**a**) Nitrogen adsorption-desorption isotherms and (**b**) the corresponding pore size distribution for CMK-3, CMK-3-H_2_SO_4_, and CMK-3-NH_3_.

Figure 1 shows XRD patterns of CMK-3, CMK-3-H_2_SO_4_, and CMK-3-NH_3_. The low-angle and high-angle patterns of XRD showed that CMK-3 and CMK-3-H_2_SO_4 _had a typical 2D-hexagonal structure, whereas the ordered mesostructure of CMK-NH_3 _was comparatively deconstructed.

**Figure 2:**
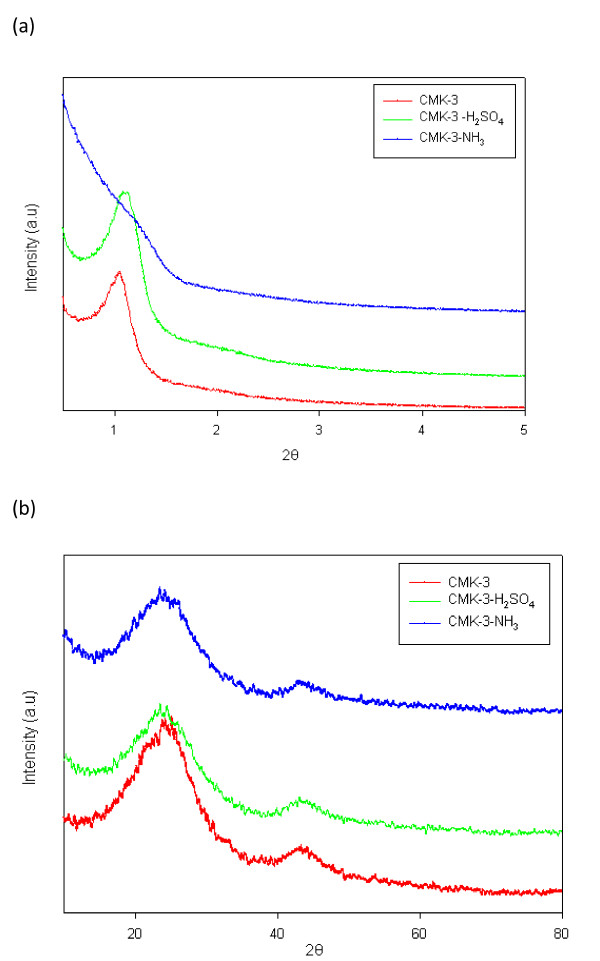
**XRD patterns of CMK-3, CMK-3-H_2_SO_4_, and CMK-3-NH_3 _samples: (a) low-angle and (b) high-angle XRD patterns**.

Table 1 lists the physical properties of mesoporous carbon used in this study. The specific surface area of CMK-3 was 1,178 m^2^/g. The specific surface area decreased slightly to 1,002 m^2^/g after a treatment with sulfuric acid, whereas it increased considerably to 1,663 m^2^/g upon a treatment with ammonia. Figure 2 shows the nitrogen sorption isotherms and pore size distributions of mesoporous carbon. Figure 2a shows the nitrogen sorption isotherms for CMK-3, CMK-3-H_2_SO_4_, and CMK-3-NH_3_. All the samples showed typical type IV isotherms of mesoporous materials under a relative pressure (P/P_0_) of 0.5. Table 1 shows that CMK-3 has mesopores with a pore size of 3.8 nm. These mesopores were maintained after a treatment with sulfuric acid. When CMK-3 was treated with ammonia, the mean pore size decreased to 3.1 nm. Treatment with ammonia is a high-temperature (900°C) process that generates micropores causing a large increase in surface area and a decrease in average pore size. On the other hand, most of the mesoporous CMK-3 were maintained after the ammonia treatment.

**Table 1:** Physicochemcial properties of various CMK-3 materials

Sample	Specific surface area (m^2^/g)	Total pore volume (cm^3^/g)	Average pore diameter (nm)	Micropore area (m^2^/g)	Micropore volume (cm^3^/g)
CMK-3	1178	1.28	3.8	142	0.05
CMK-3-H_2_SO_4_	1002	1.14	3.8	93	0.03
CMK-3-NH_3_	1633	1.55	3.1	394	0.16

Table 2 lists the elemental surface compositions obtained by XPS. CMK-3-H_2_SO_4 _showed higher C and O contents than CMK-3, whereas CMK-3-NH_3 _had lower C and O contents than CMK-3. N was detected in CMK-3-NH_3_, but not in CMK-3. This suggests that a sulfuric acid treatment and ammonia treatment have added oxygen and nitrogen functional groups, respectively, on the CMK-3 surface.

**Table 2:** Elemental surface composition of various CMK-3 materials

Sample	Atomic surface concentration obtained by XPS (%)		
	C	O	N
CMK-3	96.67	2.11	-
CMK-3-H_2_SO_4_	97.18	2.82	-
CMK-3-NH_3_	94.49	1.43	3.66

**Figure 3:**
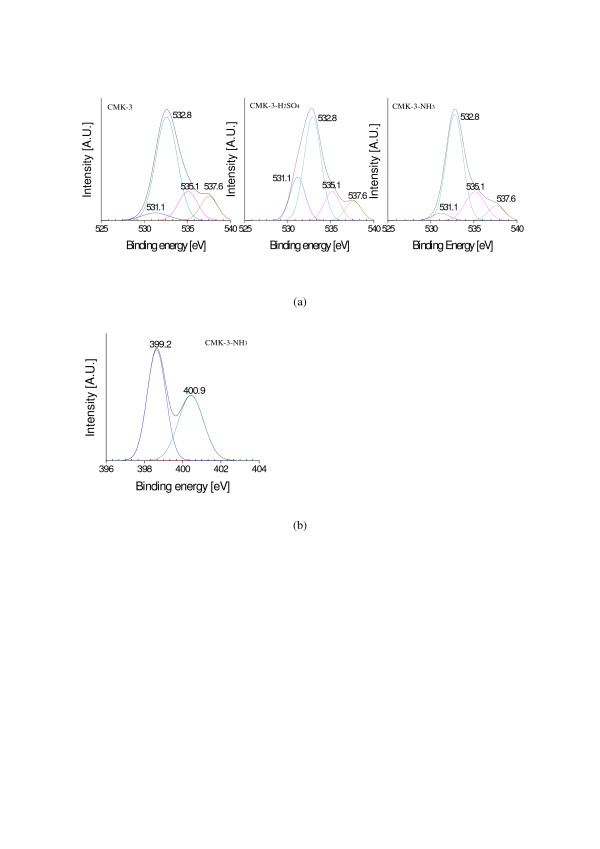
**XPS spectra of CMK-3, CMK-3-H_2_SO_4_, and CMK-3-NH_3 _samples: (a) O1s spectrum and (b) N1s spectrum**.

Figure 3 shows the O1s and N1s spectra obtained from XPS. The O1s spectra (Figure 3a) showed four peaks even though the intensity of each peak was dependent on the treatment method. Generally, the peaks appeared at the binding energy levels of 531.1 (± 0.5), 532.8 (± 0.5), 535.1 (± 0.5), and 537.6 (± 0.5) eV, representing C-OH, C=O, O_3_, and O_4_, respectively [18]. The C-OH peak was shown to increase dramatically after the sulfuric acid treatment. The N1s spectrum was detected only for CMK-3-NH_3 _(Figure 3b). N1s peaks were observed at two binding energy levels. The peaks appearing at 399.2 (± 0.5) and 400.9 (± 0.5) eV represent atomic N and pyridine-like N, respectively [19].

### Adsorption of formaldehyde

**Figure 4:**
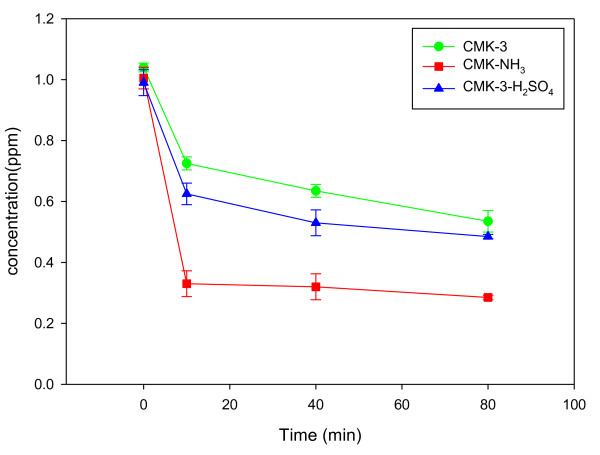
**Adsorption of formaldehyde over CMK-3, CMK-3-H_2_SO_4_, and CMK-3-NH_3 _samples**.

Figure 4 shows the results of the formaldehyde adsorption experiments using CMK-3, CMK-3-H_2_SO_4_, and CMK-3-NH_3_. Adsorption occurred most rapidly in the first 10 min and then slowed down gradually. Without treatment, CMK-3 showed a high adsorption efficiency > 50% owing to its large specific surface area. CMK-3-H_2_SO_4 _showed slightly improved adsorption performance compared to CMK-3 despite its smaller specific surface area than CMK-3, which was attributed to the highest surface O concentration resulting from an increase in oxygen functional groups. Lee et al. [20] reported that the increase in oxygen functional groups on sludge char through activation increased the formaldehyde adsorption efficiency, which is in good agreement with the present results.

CMK-3-NH_3 _showed the highest formaldehyde adsorption performance. Kim et al. [17] examined the effect of the addition of amine groups to mesoporous materials and reported that the addition of amine groups could enhance formaldehyde adsorption. Srisuda and Virote [21] also reported improved formaldehyde adsorption performance of mesoporous materials obtained by the introduction of amine groups. They argued that the interaction between formaldehyde and ammonia contributed to the increased adsorptivity. Therefore, the improved formaldehyde adsorption performance observed in the present study was attributed to the increase in nitrogen functional groups resulting from an ammonia treatment. The increase in specific surface area due to the formation of micropores might also have contributed to the increased adsorptivity.

## Conclusions

CMK-3 with a uniform pore structure, large specific surface area, large pore size, and large pore volume was applied to the adsorption of formaldehyde. CMK-H_2_SO_4 _and CMK-NH_3 _were also obtained by a treatment with sulfuric acid and ammonia, respectively. CMK-3 and CMK-3-H_2_SO_4 _had a 2D-hexagonal structure, whereas CMK-3-NH_3 _showed a somewhat disordered structure due to partial destruction of the ordered mesostructure. On the other hand, CMK-3-NH_3 _had the largest specific surface area and pore volume. XPS showed that only CMK-3-NH_3 _had nitrogen functional groups, whereas CMK-3-H_2_SO_4 _had the largest amount of oxygen functional groups. The order of the adsorption performance against formaldehyde was CMK-3-NH_3 _> CMK-3-H_2_SO_4 _> CMK-3. This was attributed to the combined effects of nitrogen and oxygen functional groups and the specific surface area.

## Abbreviations

XRD: X-ray diffraction; XPS: X-ray photoelectron spectroscopy.

## Competing interests

The authors declare that they have no competing interests.

## Authors' contributions

HBA, MJY, JMK, MJ, JKJ, SHP, SSK participated in some of the studies and in drafting the manuscript. YKP conceived of the study and participated in all experiments of this study. Also, YKP prepared and approved the final manuscript. All authors read and approved the final manuscript.
